# A Review of Ethnomedicinal Plants as Potential Anthelmintic Agents to Alternatively Control Gastrointestinal Nematodes of Ruminants in South Africa

**DOI:** 10.1155/2024/7955692

**Published:** 2024-01-16

**Authors:** Lindokuhle Christopher Mhlongo, Cresswell Mseleku, Thando Tenza, Sylvester Werekeh Fomum, Lyndy Joy McGaw, Abubeker Hassen, Ignatius Verla Nsahlai

**Affiliations:** ^1^Department of Animal Science, University of the Free State, Bloemfontein, South Africa; ^2^Department of Animal and Poultry Science, University of KwaZulu-Natal, Pietermaritzburg, South Africa; ^3^Department of Paraclinical Sciences, University of Pretoria, Onderstepoort, South Africa; ^4^Department of Animal and Wildlife Sciences, University of Pretoria, Hatfield, South Africa

## Abstract

Small ruminant production is one of the most important animal productions for food security in the world, especially in the developing world. Gastrointestinal nematode (GIN) infection is a threat to this animal's production. Conventional drugs that are used to control these parasites are losing their efficacy due to the development of resistant parasites. These drugs are not biologically degradable, taint meat products and are also expensive for communal farmers. Hence, research is now exploring ethnomedicinal anthelmintic plants for an alternative remedy. The objective of this paper was to review ethnomedicinal plants as a potential alternative to unsustainable commercial anthelmintics. This review sought to understand common GINs infecting ruminants, resistance manifestation in GINs to conventional treatment, reasons communal farmers choose ethnomedicine, and modes of action in anthelmintic plants. It also examined the usage of plants and plant parts, dosage forms, methods for improving bioactivity, convectional validation procedures, and restrictions on ethnomedicinal plant use as anthelmintics in ethnomedicine. Such insight is essential, as it highlights the importance of ethnoveterinary medicine and ways to adopt or improve it as a potential alternative to conventional anthelmintics.

## 1. Introduction

Small ruminant production is important for food security for resource-poor farmers as they are sold for cash, serve as a source of food, generate much needed income for medical needs, contribute to off-farm investments, and generate income for the purchase of additional stock [1]. A common constraint of small ruminant production is GIN infections [2]. Small-stock farmers are the ones that are affected the most by this constraint. Infection with GINs is transmitted through the consumption of infected pastures by small ruminants [3].

Conditions that increase GIN infections in ruminants are summer conditions, which favor infectious larva formation and higher grazing rates, as well as lower immunity against GINs, especially in younger ruminants [4]. Rotational grazing, anthelmintic plants, or the FAMACHA chart can all be used to control GIN infections. Moreover, vaccines such as Barbervax can be used to prevent GIN infections [5]. Unlike commercial farmers, most small stock owners do not have access to commercial anthelmintics and lack information to help manage this challenge [6]. Even when they have access to these anthelmintics, they cannot administer them correctly due to inadequate knowledge, resulting in wrong dosing [7]. These farmers are constrained to using ethnoveterinary medicinal plants exerting anthelmintic activities to control GIN in their stock. These plants are locally available to them. Ethnoveterinary medicine is the practice of controlling diseases in animals using indigenous knowledge [8]. Worldwide, commercial farmers use commercial anthelmintics, some of which include benzimidazole, imidazothiazoles, praziquantel, levamisole, ivermectin, doramectin, and moxidectin to control GINs in ruminants.

However, with the widespread development of drug resistance, these anthelmintics are becoming less effective. Resistance is defined by the lack of GIN susceptibility to anthelmintics [9]. This has left the animal production industry with a need to look for potential alternative anthelmintics. Unlike commercial anthelmintics, potential anthelmintic alternatives should be biodegradable, have no contaminants in meat, and be highly effective. Consequently, research is exploring plants with potential anthelmintic activities used by resource-poor farmers. These plants have not been sufficiently evaluated *in vitro* and *in vivo* for their anthelmintic effects, toxicity and residual effects on the host animal(s). Hence, traditional practice needs to be improved so that active natural chemicals can be identified. It is, therefore, essential to understand the common GINs that affect ruminants sheep and how resistance develops. The objective of this review is to evaluate ethnomedicinal plants with anthelmintic properties. This review discusses, but is not limited to, how parasitized small ruminants are identified and the identification of ethnomedicinal plants and their collection time, preparation methods, plant parts used, dosage, activity improvements, and limitations.

## 2. Methods

Databases of academic papers, including ScienceDirect, ResearchGate, and Google Scholar, were used to search literature for this review. We chose English-language written articles from peer-reviewed publications using “ethnoveterinary + endoparasites/gastrointestinal nematodes+ sheep/ goat / cattle,” “ethnomedicinal plants + endoparasites/gastrointestinal nematodes+ sheep/ goat / cattle,” “ethnobotany + endoparasites/gastrointestinal nematodes+ sheep/ goat / cattle,” and “traditional medicine + endoparasites/gastrointestinal nematodes+ sheep/goat/cattle” as keywords. The consideration of studies for inclusion was based on the study being a survey or review and having been conducted in sub-Saharan Africa then parasitology research papers from all over the world were used for explanations on some of the findings of the literature review.

## 3. Results

### 3.1. Gastrointestinal Nematodes of Ruminants

The presence of GIN infections causes loss of ruminant production's productivity and profit, as well as low ruminant's body condition scores in most farming communities. This results in food insecurity in the communal areas, where they depend on small ruminants in times of crop failure because of drought or inclement weather conditions. Resistant GINs are more common in small ruminants (Table 1). The most common helminths that affect ruminants belong to the nemathelminths phylum and include *Trichostrongyloidea*, *Strongyloidea*, *Metastrongyloidea*, *Ancylostomatoidea*, *Rhaditoidea*, *Trichuroidea*, *Filarioidea*, *Oxyliroidea*, *Ascaridoidea*, and *Spiruoidea* [10]. Predisposing factors for ruminants with GINs include low immunity, contaminated pastures, highly humid and wet areas, genetic make-up, overstocking of pastures, and resistance to anthelmintic drugs [11].

Ruminants are born without GIN infection but get GIN infection through grazing contaminated pasture with infective larvae (L3) [12]. Infective larvae migrate to a specific part of the gastrointestinal tract, where they grow from a preadult larva (L4) to a developed adult form (L5). Adult nematodes (male and female) live in the target site of the host (Table 1). Adult female nematodes lay 5000–10,000 eggs per day, which are passed in feces to contaminate pastures [12]. Under favorable conditions, such as a warm and moist environment, eggs hatch into larvae (L1). Thereafter, L1 larvae moult into L2 and L3 stages and accumulate in the pasture. It is known that GINs mainly feed on the erythrocytes of the host, causing compromised productivity (Tables 1 and 2) and anemia, which sometimes leads to death [11].

### 3.2. Anthelmintic Resistance by Gastrointestinal Nematodes

Resistance poses a large threat to the economic returns of ruminant farming. This is because almost all major broad-spectrum commercial anthelmintics are now ineffective against GINs [13]. Different broad-spectrum commercial anthelmintics are commonly used to control GIN infections. These drugs include benzimidazoles, imidazothiazoles, praziquantel, levamisole, ivermectin, doramectin, and moxidectin [9]. Resistance occurs when animals exposed to GINs show a decreased response to an anthelmintic drug. Similarly, resistance results when a certain population of GINs possesses a gene associated with resistance [14]. This can be due to genetic disorders such as mutation, deletion, or amplification. Furthermore, epigenetics through methylation of promoter regions or promoter regions reduces GIN's susceptibility to anthelmintics [9]. Full drug resistance is confirmed when the maximum dosage shows less efficacy [15].

Resistance manifests in two ways: decreased efficacy and delayed effectiveness of the anthelmintic against GINs. Host animals infected with drug-resistant GINs need frequent dosing compared to host animals without drug-resistant GINs. As a result, this can increase drug residues in meat products. Persistent drug resistance in ruminants is a major challenge. Hence, anthelmintic plants used in ethnoveterinary medicine are a potential alternative. This is because ethnomedicinal plants have been used for years to control GINs, with fewer reports of inefficacy. A survey reported that 79% of Ethiopian communal farmers noticed no GIN resistance, while 21% noticed GIN resistance in their anthelmintic medicinal plants [16]. The lack of resistance in anthelmintic plants might be due to the vast diversity in chemical composition as compared to commercial anthelmintics [17].

### 3.3. Reasons for Ethnomedicinal Plant Preference

Different plants are used by communal farmers to combat GIN burden in ruminants. Ethnoveterinary medicine is orally passed on from one generation to the next. Therefore, this might influence acceptance by communal farmers. Eighty percent of Africans depend on ethnoveterinary medicine to control and treat diseases in ruminants [18]. Different tribes use different ethnoveterinary medicines to treat diseases. Thus, there are a lot of anthelmintic plants available as alternatives when others become ineffective due to resistance. Communal farmers' preference of ethnoveterinary medicine over anthelminthic drugs might be because of high commercial anthelmintic cost [16], uncertainty of commercial anthelmintic's advantage over anthelmintic plants, absence of side effects, high efficacy, easy accessibility and usage, and lack of veterinarians in communal areas [19].

Most communal farmers depend on animal products, including milk and meat, but are ignorant of the drug residues in these products. Anthelmintic residues in meat and related products are a large challenge. Thus, anthelmintic remedies used to treat GINs are passed on to consumers and can be potentially harmful [20]. Synthetic drugs leave residues in hair, skin, and subcutaneous adipose tissue [21].

A survey reported that 77% of communal farmers in Ethiopia lacked knowledge about the commercial anthelmintic withdrawal period [16]. Hence, this suggests that, unlike commercial anthelmintics, ethnoveterinary practices might be beneficial to communal farmers as they do not contaminate meat products. This is because most of the medicinal plants used to treat GINs, including *C. papaya*, *A. vanbalenii*, *A. comosus*, *A. sativum*, and *A. cepa*, are edible [22]. Communal farmers were reported to prefer ethnoveterinary medicine to treat GINs in ruminants because it does not taint the meat products [23]. The fact that these treatments work well against GINs and that communal farmers believe they are better than synthetic medications are further considerations that may have contributed to their choice for ethnoveterinary medicine. It can also be the case that they are biodegradable and do not pollute the environment.

### 3.4. Diagnosis of Gastrointestinal Nematode Infection

Diagnosis of GIN infection in ruminants by communal farmers is sometimes carried out via the senses of taste, touch, smell, and sight [24]. Common signs for monitoring GIN infection in ruminants are loss of body condition, loss of appetite, and rubbing against poles [25]. Nevertheless, there are limitations that come with common signs, since helminthiasis can be confused with other diseases that have similar signs, such as fluke [23] and coccidiosis [26]. Therefore, using common signs such as body condition scores can be limited because low body weight is not a distinct sign of GIN infection [27]. Hence, there is little correlation between body condition scores and fecal egg count in terms of accurately detecting GIN infection in ruminants [28].

Adoption of these diagnostic symptoms can affect the efficacy of anthelmintics, dosage, and validity of the anthelmintic ability of the plant [19]. Distinct signs can be used to make an accurate GIN infection diagnosis because communal farmers cannot afford accurate modern methods such as the McMaster technique. One of such distinct signs of GIN parasitism is bottle jaw. This condition is caused by the depletion of blood protein when GINs suck blood from the host. However, clinical signs are not enough to diagnose GIN infection in ruminants. Hence, more reliable techniques have been developed to detect these parasites with accuracy. One of such methods for the accurate diagnosis of GIN burden in ruminants is the FAMACHA chart [28].

FAMACHA chart method identifies animals suffering from anemia, which is a common GIN infection symptom, by checking the eye color [29]. It compares the eye color of the membrane with that on the chart showing five levels of anemia. Level 1 signifies the absence of anemia, while level 5 represents a highly anemic condition [29]. Anemia is a sign of severe GIN infection by *Haemonchus contortus*. The disadvantage of this method is that anemia may be due to a nonparasitic infection [28].

Presently, fecal egg count is the most commonly used method [28]. This method uses a microscope to evaluate GINs in feces and is very accurate for detecting parasites within the host [28]. Animals with a higher nematode, egg shed, and count have the highest GIN burden. The main disadvantage of using the fecal egg count method is that communal farmers cannot adopt it without the use of a laboratory, which can be challenging for these farmers [28]. Moreover, this method does not identify the types of GINs affecting the herd. For instance, *Trichostrongylus colubriformis*, *Cooperia* spp., and *Bunostomum trigonocephalum* highly affect sheep; on the other hand, *Oesophagostomum columbianum* and *Haemonchus contortus* affect goats [30].

There are two types of fecal egg count tests, one of which is qualitative and the other is quantitative. A qualitative test is the floatation of contaminated fecal samples under a microscope to examine GINs [28]. The results are reported as positive or negative as proof of infection progress over time [28]. A quantitative test uses eggs per gram of known weight of a sample of feces, a McMaster slide, and floatation solution. Two chambers of the slide are filled with fecal solution multiplied by the dilution factor, and the type of nematode eggs is identified under the microscope [28]. The quantitative evaluation technique is easier, more inexpensive, and more reusable compared to the qualitative fecal egg count method [28].

### 3.5. Modes of Action for Anthelmintic Ethnomedicinal Plants

**Figure 1:**
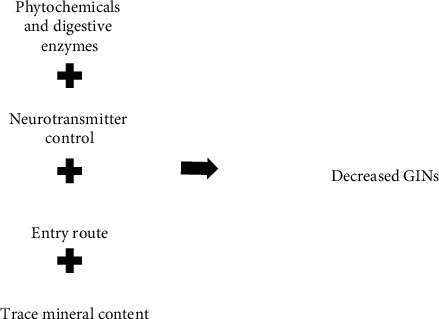
Mode of action's factors for ethnomedicinal plants with anthelmintic activity.

Anthelmintic medicinal plants are believed to have various strategies of controlling GINs which are listed below (Figure 1). It is believed that the effect of anthelmintic medicinal plants on GINs depends on phytochemical and trace mineral content, GIN neurotransmitter control, and entry routes used by ethnomedical plants to penetrate GINs.

#### 3.5.1. Phytochemicals and Digestive Enzymes

Different plants, used in ethnoveterinary medicine to control GINs, contain different anthelmintic phytochemicals and enzymes (Table 3). It is not fully known how all phytochemicals of different plants used by communal farmers control GINs, except for a few like those mentioned in Table 4. Plants like papaya and fig trees have latex, which contains a lot of proteolytic enzymes, while pineapples have cysteine proteinases. These enzymes digest GINs. *Ficus* spp. has also been reported to have ficin [31]. *Saba senegalensis* has compounds such as tannins, saponins, triter, pene glycoside, and steroids. These compounds attach to free proteins within tubes for larval nutrition, thus killing the GINs [32]. While commercial anthelmintics contain one molecule acting on the parasite(s), anthelmintic plants possess numerous active molecules that act together in synergy against gastrointestinal parasites. This increases efficiency and reduces the development of resistant GINs [33]. Aloe has amino acids such as sterols and phenols, which negatively affect the protein and body repair of GINs [34]. While ginger's anthelmintic activity is due to gingerols, shogaols, zingerone, and paradol [35], these phytochemicals activate cholinergic receptors. This causes a contraction of the gastrointestinal tract, which expels parasites [36].

#### 3.5.2. Neurotransmitter Control

Active phytochemical in an anthelmintic plant extract is the one with the ability to inhibit acetylcholinesterase of GINs [37]. Acetylcholinesterase is a serine hydrolase that is responsible for the catalysis of a neurotransmitter called acetylcholine into acetate and choline. This results in the formation of a substrate-enzyme complex. This is followed by acetylation of the hydroxyl group of the amino acid serine, which is present in the esteratic site that is finally deacetylated. Its inhibition leads to paralysis and death of the GINs [38].

*Helicotylenchus dihystera* treated with *Punica granatum*, *Thymus vulgaris*, and *Artemisia absinthium* extracts were reported for suppression of acetylcholine in nematodes [37]. It was then concluded that the efficacy of these extracts shows a relation between nematode poisoning and the inhibition of acetylcholine. This suggests that the observed efficacy of the used plant extracts is partly due to the inhibition of acetylcholine activity.

#### 3.5.3. Entry Route

Anthelmintic drugs penetrate GINs commonly via oral access or transcuticular diffusion. It is argued that the latter route is the most common way of entry for anthelmintic drugs in GINs [39]. Hence, an effective extract against GINs must have phytochemicals that can penetrate the cuticle of GINs [39]. Lipophilic anthelmintics exert their effects through transcuticular diffusion easily compared to hydrophobic ones [40]. This suggests that the extract type that is more effective might contain more lipophilic than hydrophilic chemicals.

#### 3.5.4. Trace Mineral Content

For reducing GIN infection, taking supplements of trace minerals (iron, zinc and copper) boosts immunity, particularly at critical physiological periods [41]. This is due to a positive correlation between white blood cells and trace mineral content [42]. Presence of trace minerals can improve plant extract efficacy against GINs. In a previous study, efficacious anthelmintic extracts tended to have a high content of zinc, copper, and protein in addition to flavonoids and tannins [43]. Supplementation with copper kills GINs and decreases egg counts [44]. Hence, nutritious plants with high trace minerals and anthelmintic phytochemical content are potential alternative pastures. Such types of pastures can eliminate the need for laborious vaccination and harvesting of anthelmintic plants.

### 3.6. Common Anthelmintic Ethnomedicinal Plants

Table 4 shows that there is a wide range of plant families that are used for anthelmintic ethnomedicinal medicine. Previous surveys reported that communal farmers predominantly use plants of the Fabaceae family [45–47]. Other studies differ; *Asphoedelaceae* was the most frequently used plant family by communal farmers in Kwezi and Ntambethemba villages in Eastern Cape province [19]. Respondents in Nhema village, Zimbabwe, frequently used plants of the families *Fabaceae*, *Solanaceae*, and *Asphoedelaceae* [26].

The use of different families of plant species in various regions seems to be influenced by plant population distribution and their multiple biological activities [26]. This is exemplified by plant species including *Clerodendrum glaum*, which is used in treating helminths, diarrhea, bile, and cough, while *Gnidia kraussiana* is used in treating bile and cough in addition to its anthelmintic activity. Besides their anthelmintic activity, *Laportea peduncularis*, on the other hand, is used to treat diarrhea and cough; *Salvadora australis* is used to treat foam in cattle; and *Ziziphus mucronata* also treats diarrhea [48]. Similarly, *Zingiber officinale* is used in ethnoveterinary medicine to treat arthritis, rheumatism, sprains, muscular aches, pains, sore throats, cramps, indigestion, nausea, hypertension, dementia, and fever in addition to treating GINs [49]. Plant species families that can treat human or multiple livestock diseases in addition to GINs of livestock seem to be prioritized.

### 3.7. Anthelmintic Ethnomedicinal Plant's Preparation

It is believed that ethnomedicinal plant parts should be harvested under certain conditions for the preparation of ethnomedicinal plants that sufficiently control GINs. Because they continuously store phytochemicals, roots are picked every year; bark is harvested while sap is running; and fruit and seeds are harvested early in the fruit ripening season [50]. Leaves are collected before the flowering season, as plants use metabolites for flowering. Leaves are usually collected in the summer [24] since there is a supply of effective medicinal plants [18], as phytochemicals peak at this time. To avoid harvesting during a peak GIN infection [51], the harvest period must be during the off-peak GIN infection period to avoid contamination of ethnomedicinal plants with GINs. The advantage of this is that peak anthelmintic phytochemicals coincide with winter, an off-peak GIN infection season. For example, tannin content, a common anthelmintic phytochemical, increases during winter but decreases during summer months [52].

Ethnoveterinary medicinal plants with anthelmintic activities can be prepared through boiling and infusion. Table 4 shows that boiling (aqueous solution) is the most commonly used method of preparation of anthelmintic plant species per plant part by communal farmers [19, 25, 48]. Boiling is suggested to either deactivate toxic thermolabile components of plants that can be poisonous to the GIN infected animal [19] or deactivating some of the active anthelmintic phytochemicals that are thermolabile [19]. Water may dilute the concentration of plant extracts and render crude extracts less poisonous [8]. The choice of aqueous extracts by communal farmers might be because water is easily accessible. This method of preparation is also easy to master, as it only requires water to boil for a certain time.

The solvent used seems to influence the efficacy of anthelmintic plant extracts. Aqueous plant extracts, which are a common extract type, have lower efficacy compared to ethanolic and methanolic plant extracts [33]. This might be due to aqueous extract characteristics such as low anthelmintic activity, biological activity, and type of phytochemicals [45, 53]. Differences between aqueous and other types of extracts might be attributed to the different proportions of phytochemicals extracted by solvent types resulting in different effects on GINs [39]. Therefore, water might be extracting fewer phytochemicals compared to other solvents such as ethanol, acetone, chloroform, and methanol due to being used to extract phytochemicals from plants containing less water extractable phytochemicals.

*Iris kashmiriana* aqueous extract from sheep showed superior *in vitro* efficacy against *Haemonchus contortus* compared to methanolic extracts (100 vs. 85%, respectively) [54]. In the same study using the *in vivo* method, the aqueous extract remained superior compared to the methanolic extract (70.2 vs. 33.2%, respectively). The superiority of aqueous extracts was explained to be due to the high concentration of water-soluble active molecules within the extracts. Aqueous plant extract effect on GINs can also be expected to increase with immediate use after preparation while fresh since aqueous anthelmintic plant extracts have a low shelf life because water allows microbial growth [55]. There is a need for a solvent that can extract polar and nonpolar anthelmintic phytochemicals. This is because the plant cell contains water-soluble and non-water-soluble bioactive chemicals. Therefore, a mixture of water and other solvents can be used for extraction for improved efficacy against GINs. For instance, aqueous-ethanol is a better solvent than pure ethanol because a proportion of bioactive chemicals are polar or nonpolar and can be extracted to increase plant extract efficacy [54]. Other types of solvents can be used for plant extraction; acetone extracts for both hydrophilic and lipophilic phytochemicals in plants, which is useful especially when phenolic plants need to be extracted [54]. Moreover, ether is better suited for the extraction of fatty acids and coumarin compounds from the plant's anthelmintic activity [54]. Similarly, chloroform is also better at extracting terpenoids and lactones.

### 3.8. Common Plant Parts Used in Anthelmintic Ethnomedicinal Plants

Commonly used plant parts to control GINs are the leaves of anthelminthic plants (Table 4) [19, 26]. This might be because leaves are infective larvae-free since they are at the top of trees and dry compared to roots and bark, which can be close to contaminated grass. Communal farmers were found to prefer using leaves because harvesting them is easier compared to collecting other plant parts [47]. The other reason communal farmers prefer leaves is because they want to conserve the plants to avoid extinction, as opposed to using roots or stems [8, 19]. Consequently, picking leaves for ethnoveterinary medicine tends to lead to plant extinction, especially if the leaves picked are the younger ones instead of the old ones since leaves are biologically important for the survival of plants [56]. On the other hand, some communal farmers use the whole plant to prepare medicine because they believe it increases efficacy [57]. It is also suggested that plant parts with a higher shelf life are highly preferred, such as bark, bulbs, fruits, and seeds [58] and shrubs, tubers, and whole plants [26].

One survey reported that 58.3% of farmers in the Benoue region of Cameroon were commonly using stem and bark [25], perhaps the trees used are in abundance. This suggests that different plant parts have different levels of activity in combatting GINs in ruminants. These commonly used plant part(s) might be chosen based on relative efficacy to other plant parts. For instance, pineapple has more anthelmintic phytochemical(s) (bromelain) in the stem compared to other parts and will most likely exert greater efficacy if used as a source of extract.

Thus, this suggests that plant parts with a higher proportion of phytochemicals than others should be isolated and used to control GIN in small ruminants. This is because the leaves are part of the browse that goats feed on. Hence, GINs of goats might be adapted to phytochemicals within the browse. Goats also seem to acquire a weak immune system towards GINs as goat GINs develop resistance quicker compared to sheep GINs [53]. Therefore, anthelmintic plants which are effective in goats are also expected to be more effective in sheep against GINs.

### 3.9. Dosages of Anthelmintic Ethnomedicinal Plant Extract

Ethnoveterinary medicine doses used to control GINs are measured using spoons, calabash bottles, clay pots, hand palms, and finger pinches [24]. Sometimes, qualitative measures determine concentrations, such as color change, once the plant material is soaked in water [24]. As a result, most ethnoveterinary medicines may be toxic compared to modern anthelmintics [17]. Therefore, there is no exact amount of plant material per volume of water that is suitable to effectively treat GINs without adding negative effects on the GIN-infected ruminants. Hence, ethnoveterinary medicine needs to be standardized for effective concentration, as this can prevent underdosing or overdosing [23]. Thus, standardization of ethnoveterinary medicine concentration can limit the death of ruminants from toxicity and residues in meat products. For instance, treatment of GINs by ethnoveterinary medicine focuses more on plant extract volume than phytochemical concentration per volume of plant extract. For example, it is reported that the dosing of plant extracts is limited to using 0.75–1 L cold drink bottles per animal without being precise about the phytochemical concentration, which may lead to under- or over-dosing of ethnoveterinary medicine [59].

### 3.10. Improvement of Anthelmintic Activity in Ethnomedicinal Plant Extracts

Different anthelmintic plants are mixed by communal farmers with one another for synergistic purposes or with other nonplant substances to increase the efficacy of treatment. These nonplant substances used include flour (laxative effect), butter (increased flavour), rock salt (emulsification), oil cake (labile secretion), and Epsom salt [19]. Therefore, to prevent inconsistent anthelmintic activities, studies need to determine whether plant extracts work best individually or in combination [60]. Some plant combinations are synergistic when the dose ratio is different between plants involved in the combination. It has been found that the synergistic effect tends to happen at lower concentration of tannin types in flavonoids and condensed tannin combination [60].

Combination of *Vernonia anthelmintica* and *Embelia ribes* have also shown 83-93% efficacy in controlling the GINs [61]. Synergism defines a condition where two or more agents are combined to result in an effect that is greater than that of a single agent [60]. Synergistic effect is calculated by monitoring additive individual effects from treatments [62]. They are then compared to effects from a combination of treatments on the assumption that they have independent effects. The additive effect is compared with the combination effect of treatment. If the additive effect is less than the combined effect, then there is synergism, while if it is more than a combined effect, then there is antagonism [63]. Synergism is advantageous because that is where a plant combination which is effective in both sheep and goats can be identified, since these plants produce different anthelmintic activities in these ruminants [22].

There is also the advantage of discovering a combination of plants with phytochemicals that can combat resistance by targeting different GIN species. This is due to different plant extracts being specific on GIN species and growth stage [64]. For instance, Fagara extracts have been shown to specifically affect GIN eggs and adult GINs [65]. Therefore, this suggests that combining different plant extracts can enhance their broad-spectrum effects on GIN species and growth stages since GIN-infected ruminants contain GINs of various parasitic growth stages and species.

### 3.11. Anthelmintic Activity Validation in Ethnomedicinal Plants

Out of 250 000 plant species in the world, only 4–5% have been studied for bioactive chemicals [66]. Therefore, most anthelmintic plants still need to be discovered and studied for their anthelmintic activities [66]. This is because these plants have different phytochemical compositions that produce different anthelmintic activities [66]. Bioassays used for isolating plants with anthelmintic activities should be simple, accurate, and affordable. This is vital for the easy identification of small concentrations of effective and ineffective compounds [66]. The *in vitro* method is the most commonly used bioassay to isolate anthelmintic plants because it is ethical, less laborious, and cheap [66]. This study has a laboratory imitation of biological conditions without using an animal. *In vivo* studies involve feeding a parasitized host animal a certain amount of anthelmintic plants [46]. *In vivo* studies produce more accurate results than *in vitro* studies, but due to animal welfare rules in many countries, they have limited use [66].

*In vitro*, tanniferous plant extracts affected GIN burden [22]; however, *in vivo*, the effect on GIN was absent [52]. This might be attributed to the change in anthelmintic properties of the plant by the gut microorganisms in the gastrointestinal tract of the host [42]. To counter the *in vivo* inactivation of plant extracts, communal farmers concentrate extracts to increase the number of compounds prone to inactivation by ruminal microorganisms [7]. Thus, *in vivo*, there is a need to identify plant extracts with phytochemicals that are resistant to digestion by the microflora of the gut as concentrating plant extracts can potentially lead to the poisoning of ruminants. However, the disadvantage of *in vivo* bioassay is that it uses few control animals. Hence, this inhibits the statistical analysis of data because it is expensive and labor-intensive. In addition, *in vivo* methods have a lot of indirect and direct factors that influence the results, such as nutrition, age, and season, but it is the most useful method of anthelmintic plant validation.

### 3.12. Limitations of Anthelmintic Plants as Alternatives to Conventional Products

The use of plants as an alternative control for GINs in ruminants is limited by their mysterious compounds and direct and indirect mechanisms of action on parasites and hosts. There is also a lack of a conventional preparation method for these plants [66]. This is because it is difficult to prepare ethnomedicine as communal farmers do [66]. Hence, using bioassays such as *in vitro* and *in vivo* might exaggerate the efficacy of ethnomedicinal plants [66]. Thus, the adoption of an incorrect dose is possible. As a result, this might increase the toxicity of these plants. Toxicity increases with efficacy due to dose dependence. Hence, an efficacious dose might be too toxic to be used in animals [66] to obtain the desired efficacy.

Other factors, such as ease of plant cultivation, harvesting, supply, and mode of administration, can limit the use of plant extracts. Palatability, stability, biodegradation of anthelmintic compounds within the plants, and lack of accurate dosage can also lead to the poisoning of animals [67]. Some plants, such as *Lotus* spp. and *H. coronarium*, are weak, cannot tolerate grazing and stamping by ruminants, and die easily [67]. Furthermore, communal farmers tend to give a single collective name to a group of plants based on their resemblance or characteristics. Plants producing latex are collectively called Mithuri by Kenyan communal farmers, regardless of plant family or medical purpose [56], which makes anthelmintic plant identification difficult. Traditional healers are also very secretive about ethnoveterinary medicine. This limits the identification of most anthelmintic plants [17]. Communal farmers in the Nhema midlands of Zimbabwe explained that the reason for secrecy about ethnoveterinary medicine knowledge is jealousy among custodians of this practice [19]. Secrecy might also be because this knowledge is passed down orally and strictly through family lineages [56]. Furthermore, herbalists use plants of different efficacies to make ethnoveterinary medicines. Customers tend to prefer the herbalist with the most effective ethnoveterinary medicine. Therefore, the explanation for secrecy might be the competition between herbalists to attract more customers. This limits the discovery of plants with effective compounds because full information is not given [59].

## 4. Conclusion

As they are working for communal farmers, ethnomedicinal plants are a potential source of ingredients to develop sustainable commercial anthelmintics. However, their activity is anecdotal, as it is not standardized for scientific use. Therefore, to produce safe, effective, and noncontaminating anthelmintics, these plants need to be further evaluated scientifically following an *in vitro* assay. Then, they can be used to treat animals. Studies should focus on their toxicity, dose response, chemical composition, mode of action, and synergism to produce a reputable source of anthelmintics.

## Figures and Tables

**Table 1:** Gastrointestinal site of infection per different gastrointestinal nematodes that infect ruminants.

Oesophagus and omasum	Abomasum	Small intestines	Large intestines
*Cotylophoron* spp, *Gongylonema pulchrum*, and *Paramphistomum* spp.	*Haemonchus contortus*, *Teladorsagia circumcincta*, *Teladorsagia trifurcata*, *Parabonema* spp., and *Trichostrongylus axei*	*Avitellina centripunctata*, *Bunostomum trigonocephalum*, *Cooperia curticei*, *Cooperia surnabada*, *Gaigeria pachyscelis*, *Moniezia expansa*, *Nematodirus battus*, *Nematodirus filicollis*, *Nematodirus spathiger*, *Strongyloides papillosus*, *Trichostrongylus capricola*, and *Trichostrongylus vitrinus*	*Chabertia ovina*, *Oesophagostomum columbianum*, *Oesophagostomum venulosum*, *Skrjabinema ovis*, *Trichuris ovis*, and *Trichuris skrjabini*
[12]	[12]	[12]	[12]

Information contained per column is from the reference placed below each column.

**Table 2:** Characteristics of different gastrointestinal nematodes that infect ruminants.

Scientific (common) name	Morphology	Prepatent period	Signs	Source
*Haemonchus contortus* (barber-pole)	Length, 10-30 mm; white uteri and ovaries; barber-pole look	18-22 d	Acute anemia, intense blood loss, bottle jaw, stool, pale gums, and inner eyelids.	[12]

*Nematodirus* spp. (thread-necked strongyle)	Length, 10-30 mm; thin exterior; swollen head	15-28 d	Inappetence, stool, weight, and wool loss.	[12]

*Trichostrongylus* spp. (bankrupt worm/stomach hairworm)	No filament	20-25 d	Weight loss, reduced growth rate, inflammations, stool, and inappetence	[12]

*Cooperia* spp. (small intestine worm)	Length; 4-6 mm; brownish-red	15-20 d	Inappetence, stool, and weight loss.	[12]

*Oesophagostomum* spp. (nodular worm)	Length, 20 mm; thin front	6-7 d	Stool, swelling large intestinal wall, and mucus-covered feces.	[12]

*Trichuris* spp. (whipworm)	Length, 35-80 mm; thin neck; thick hind end; curved tail	1-3 months	Caecal wall swelling and stool.	[12]

**Table 3:** Different anthelmintic phytochemicals found in plant extracts and their effect on gastrointestinal nematodes that infect ruminants.

Anthelmintic phytochemicals	Mode of action
Saponins	Targets the permeability of the cuticle of the parasites.
Benzyl isothiocyanate	Paralyses the motor activity and metabolism of the parasite.
Cysteine proteinases	Contains proteolytic chymopapain and papain, which are responsible for the breakdown of the parasites' cuticle.
Isoflavones	Affects the glycolysis and glycogenolysis activity enzymes and calcium ions of the parasite.
Artemisinin	Causes the cleavage of endoperoxide bridges by iron-producing free radicals. This stresses the biological molecules of the parasite through oxidation.
Phenolic compounds	Uncouple the oxidative phosphorylation mechanism and disturb the glycoprotein of the cell surface, resulting in the death of the parasite.
Tannins	Uncouple the oxidative phosphorylation, attach to free glycoproteins of the gastrointestinal wall, and attach to the glycoproteins of the parasites causing death to the parasite.
Alkaloids	Paralyse the central nervous system, steroidal alkaloids, and oligoglycosides which suppress sucrose from travelling from the stomach to the small intestines; alkaloids act as an antioxidant, thus inhibiting homeostasis condition excellent for parasite development.
[12]	[12]

Information contained per column is from the reference placed below each column.

**Table 4:** Anthelmintic plant species used by South African communal farmers to control ruminant's gastrointestinal nematodes.

Plant family	Scientific (common) name	Plant part	Preparation	Reference
Apocynaceae	*Acokanthera oppositifolia* (Bushman's poison)	Leaves	Boiling	[68]
Apocynaceae	*Dichrostachys cinerea* (sickle bush)	Leaves	Boiling	[48]
Apocynaceae	*Salvadora australis* (mustard tree)	Leaves	Boiling	[48]
Agapanthaceae	*Agapanthus praecox* (African lily)	Leaves	Infusion	[68]
Amaryllidaceae	*Crinum macowanii* (Cape coast lily)	Leaves	Boiling	[48]
Anacardiaceae	*Harpephyllum caffrum* (wild plum)	Bark	Boiling	[68]
Apiaceae	*Centella coriacea* (swamp pennywort)	Bark	Boiling	[68]
Araliaceae	*Cussonia spicata* (Natal cabbage tree)	Bark	Infusion	[68]
Asphodelaceae	*Aloe ferox* (bitter aloe)	Leaves	Boiling	[68]
Asphodelaceae	*Gasteria bicolor* (elephant's Foot)	Leaves	Infusion	[68]
Asphodelaceae	*Bulbine latifolia* (broad-leaved bulbine)	Leaves	Boiling	[68]
Asphodelaceae	*Bulbine frutescens* (cat's tail)	Whole plant,	Infusion	[68]
Asphodelaceae	*Bulbine abyssinica* (snake flower)	Leaves	Boiling	[68]
Asphoedelaceae	*Aloe arborescens* (bitter aloe)	Leaves	Boiling	[68]
Asteraceae	*Vernonia neocorymbosa* (Vernonia)	Leaves	Boiling	[47]
Bignoniaceae	*Kigelia africana* (sausage tree)	Leaves	Boiling	[48]
Capparidaceae	*Capparis sepiaria* (caper bush)	Roots	Infusion	[68]
Euphorbiaceae	*Ricinus communis* (castor bean)	Leaves	Boiling	[68]
Fabaceae	*Elephantorrhiza elephantina* (elephant's root)	Roots	Boiling	[48]
Fabaceae	*Schotia latifolia* (bush boer bean)	Bark	Boiling	[19, 68]
Fabaceae	*Erythrina caffra* (coral tree)	Leaves	Boiling	[68]
Geraniaceae	*Pelargonium reniforme* (Pelargonium)	Tuber	Boiling	[68]
Gunneraceae	*Gunnera perpensa* (river pumpkin)	Tuber	Boiling	[48]
Hyacinthaceae	*Albuca setosa* (soldier in the box)	Tuber,	Boiling	[68]
Hypoxidaceae	*Hypoxis argentea* (yellow stars)	Tuber	Boiling	[19]
Lamiaceae	*Teucrium trifidum* (Dutchmen's fever plant)	Leaves	Infusion	[68]
Lamiaceae	*Leonotis leonurus* (wild dagga)	Leaves	Boiling	[68]
Lamiaceae	*Ocotea bullata* (black stinkwood)	Bark	Boiling	[68]
Loganiaceae	*Strychnos henningsii* (red bitter berry)	Bark	Boiling	[68]
Moraceae	*Ficus ingens* (fig tree)	Leaves	Boiling	[48]
Pittosporaceae	*Pittosporum viridiflorum* (cheese wood)	Bark	Infusion	[48]
Polygonaceae	*Rumex lanceolatus* (common dock)	Roots	Boiling	[48]
Ptaeroxylaceae	*Ptaeroxylon obliquum* (sneeze wood)	Leaves	Boiling	[48]
Rhamnaceae	*Ziziphus mucronata* (buffalo thorn)	Leaves	Infusion	[68]
Rutaceae	*Zanthoxylum capense* (small knob wood)	Roots	Boiling	[68]
Sterculiaceae	*Hermannia incana* (sweet yellow bells)	Whole plant	Boiling	[68]
Thymelaeaceae	*Gnidia kraussiana* (wellow heads)	Leaves	Boiling	[48]
Tiliaceae	*Grewia occidentalis* (cross berry)	Bark	Boiling	[68]
Urticaceae	*Laportea peduncularis* (river nettle)	Leaves	Boiling	[48]
